# Multi-Layered Branched Surface Fluorination on PVDF Membrane for Anti-Scaling Membrane Distillation

**DOI:** 10.3390/membranes12080743

**Published:** 2022-07-29

**Authors:** Yu-Jing Liu, Yan-Nan Lu, Dong-Qing Liang, Yin-Shuang Hu, Yu-Xi Huang

**Affiliations:** 1 School of Environmental Science and Engineering, Sun Yat-sen University, Guangzhou 510275, China; liuyj225@mail2.sysu.edu.cn (Y.-J.L.); luyn7@mail2.sysu.edu.cn (Y.-N.L.); liangdq6@mail2.sysu.edu.cn (D.-Q.L.); huysh9@mail2.sysu.edu.cn (Y.-S.H.); 2 Guangdong Provincial Key Laboratory of Environmental Pollution Control and Remediation Technology, Sun Yat-sen University, Guangzhou 510275, China

**Keywords:** membrane distillation, multi-layered surface modification, scaling resistance, superhydrophobic membrane

## Abstract

Membrane distillation (MD) has emerged as a promising technology for hypersaline wastewater treatment. However, membrane scaling is still a critical issue for common hydrophobic MD membranes. Herein, we report a multi-layered surface modification strategy on the commercial polyvinylidene fluoride (PVDF) membrane via plasma treatment and surface fluorination cycles. The repeated plasma treatment process generates more reaction sites for the fluorination reaction, leading to higher fluorination density and more branched structures. MD tests with CaSO_4_ as the scaling agent show that the modification strategy mentioned above improves the membrane scaling resistance. Notably, the PVDF membrane treated with three cycles of plasma and fluorination treatments exhibits the best anti-scaling performance while maintaining almost the same membrane flux as the unmodified PVDF membrane. This study suggests that a highly branched surface molecular structure with low surface energy benefits the MD process in both membrane flux and scaling resistance. Besides, our research demonstrates a universal and facile approach for membrane treatment to improve membrane scaling resistance.

## 1. Introduction

Attributed to the global water shortages and environmental concerns brought by hypersaline brine discharge [1,2,3], membrane distillation (MD) emerged as a promising membrane-based thermal desalination technology in the late 1960s [4,5]. Unlike reverse osmosis (RO) and conventional thermal distillation, MD has unique advantages of low capital cost, high selectivity, mild operating conditions, and low energy demand [3,4,5]. MD is capable of desalinating seawater, brackish water, shale gas-produced water, and concentrated RO brine for zero liquid discharge (ZLD) [2,6,7,8] using low-grade thermal energy like waste heat, geothermal, or solar energy [4,9]. As a physical barrier that separates the feed and distillate solutions [9,10,11,12], a hydrophobic microporous membrane is indispensable during the MD process. Currently, commercial hydrophobic membranes such as polyvinylidene fluoride (PVDF), polypropylene (PP), and polytetrafluoroethylene (PTFE) are widely employed in MD [10,13,14,15]. However, traditional MD membranes are subject to membrane fouling, scaling, and pore wetting [2,15,16], which are highly associated with the membrane properties and the operating conditions [12,17,18]. When treating inorganic hypersaline brine, scale deposits such as CaCO_3_ and CaSO_4_ on the membrane surface directly link to the membrane scaling, wetting, and/or blocking [6,9,19]. Therefore, it is critical to address the above issues for the MD applications. 

Various strategies have been developed to modify the membrane wettability for better MD performance [7,9,14,20]. Membranes with superhydrophobicity can mitigate heterogeneous nucleation or scale deposition on the membrane surface, exhibiting remarkable scaling and wetting resistance [7,14,21]. A low surface energy composite with a multiscale re-entrant structure is generally adopted to achieve superhydrophobicity [5,16]. This membrane preparation strategy usually includes the addition of other substances like dopamine (DA) and polyethyleneimine (PEI) [3], incorporating nanoparticles such as SiO_2_ [14,15,16,17,18], TiO_2_ [19], activated carbon (AC) [20,21], immobilizing carbon nanotubes (CNTs) [11,22,23,24,25], surface corrugation via imprinting technique or double layer [26,27,28], and surface fluorination [10,29]. Among them, grafting nanoparticles on hydrophobic membrane substrates is the most effective way. However, the use of nanoparticles commonly leads to a high degree of surface roughness [18,30], thereby increasing the mass transfer resistance and impacting the water vapor flux of MD [10,16]. A recent study has proved that coating a liquid-like layer of fluorinated molecules on a PVDF membrane without adding nanoparticles could improve the membrane scaling resistance while maintaining uncompromised vapor flux [29]. This finding suggests that a liquid-like fluorosilane coating could affect the membrane surface hydrodynamic conditions and lower the surface energy, thereby improving the scaling resistance without sacrificing the flux. However, how the coating density and/or the molecular chain branch level affect the MD scaling performance remains unknown.

Based on these concerns, this research focuses on enhancing the anti-scaling capability of commercial PVDF membrane, which is universally used in the MD process. We propose a membrane modification strategy that combines plasma treatment and chemical vapor deposition (CVD) of fluorosilane. The plasma treatment aims to generate hydroxyl groups on the membrane surface, while the CVD process aims to graft the fluorosilane with the hydroxyl groups. We hypothesize that repeating the plasma-CVD treatment cycle may increase the density of the fluorosilane coating. In addition, the repeated plasma treatment may also generate branch structure on the membrane surface, which could further impact the surface wettability. Herein, membranes prepared using the above plasma-CVD strategy are systematically studied. The morphologies and surface properties of these membranes are characterized and compared to reveal the effect of the plasma-CVD treatment cycle. MD tests are conducted to investigate the vapor flux and anti-scaling performance of the prepared membranes. 

## 2. Material and Method

### 2.1. Chemicals and Materials

Commercial polyvinylidene fluoride (PVDF, Durapore^®^ Membrane Filters, 0.22 μm GVHP00010) membrane was purchased from Merck Millipore (Shanghai, China). *N,N*-dimethylformamide (DMF, AR, >99.9%) was purchased from Aladdin (Shanghai, China). Sodium sulfate (AR, Na_2_SO_4_), calcium chloride dihydrate (AR, CaCl_2_·2H_2_O), and sodium chloride (AR, NaCl) were purchased from Guangzhou Chemical Reagent Factory, Guangzhou, China. 1*H*,1*H*,2*H*,2*H*-perfluorodecyldimethylchlorosilane (C_12_H_10_ClF_17_Si, 90%) (17-FAS-S) was purchase from Alfa Aesar (Shanghai, China). Deionized (DI) water from a Millipore Milli-Q water system (Shanghai, China) was used. All chemicals were used as received.

### 2.2. Membrane Surface Modifications

There were two steps of surface modification for the commercial PVDF membrane. The first step was to functionalize the surface with hydroxyl groups using a plasma treatment machine (SANHOPTT PT-10ST, Shenzhen, China) under −100 kPa vacuum for 10 min. The second one was to fluorinate the surface of the treated membrane through CVD. In this step, the plasma-treated membrane was sealed in a box, together with a solution consisting of 240 μL DMF and 60 μL FAS. Then the box was put into a vacuum oven at 90 °C for 12 h. During the surface fluorination, the membrane was placed on a custom-built stage in the sealed box to prevent it from directly contacting the DMF/FAS solution. Finally, all the membranes processed this way were dried at 90 °C to remove the residual solvent. 

**Figure 1:**
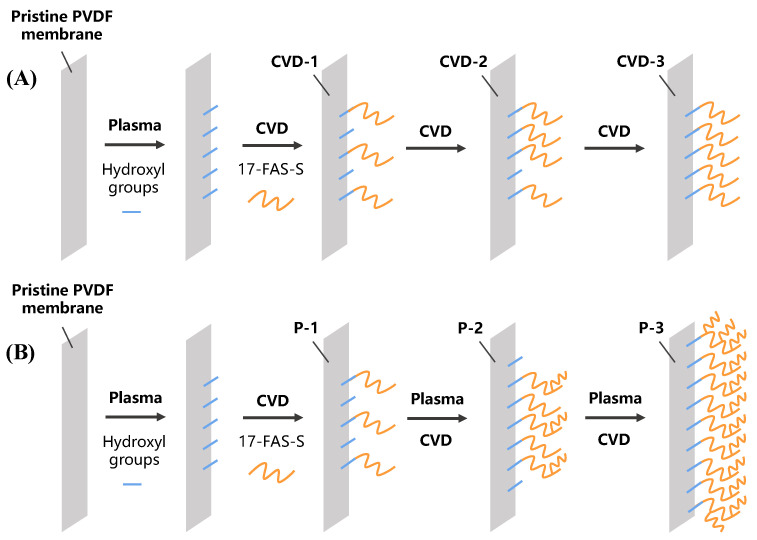
The procedure of repeated plasma-CVD modification strategies. (**A**) Modification process with multiple CVD steps and (**B**) modification process with multiple plasma-CVD cycles.

The plasma treatment power was firstly optimized in our preliminary experiment. Three powers of plasma treatment were employed in the first step, including 50 W, 100 W, and 150 W, respectively. After the same second step, the membrane properties and MD performances were tested to find the best treatment power. Two modification strategies were formulated based on the optimal power to increase the coating density. The first approach contained one plasma treatment, followed by one, two, or three times CVD (Figure 1A). Membranes prepared in the first approach were denoted as CVD-1, -2, and -3, respectively. The second approach repeated the plasma-CVD cycle one, two, or three times (Figure 1B). Membranes prepared in the second approach were denoted as P-1, -2, and -3, respectively. It should be noted that the CVD-1 and P-1 were the same.

### 2.3. Characterizations

Pore size distribution was tested by a Porometer CFP-1500-LP (PMI, Peachtree Corners, GA, USA). The static water contact angle was measured by a Drop Shape Analyzer (Krüss, DSA25E, Hamburg, Germany). The gravimetric method was used to measure the porosity of the membranes. The membrane surface morphology was inspected with a scanning electron microscope (SEM, Quanta 400 FEG, Hillsboro, OR, USA). The SEM was equipped with an energy-dispersive X-ray spectroscopy detector (EDS) that provided the elemental mapping of the initial and scaled membrane samples. The element chemical states of the membrane surfaces were analyzed by an X-ray photoelectron spectrometer (XPS) (ESCALab250, Thermo Fisher Scientific, Waltham, MA, USA) equipped with an Al Kα excitation source. 

### 2.4. Membrane Distillation Experiments

The anti-scaling and anti-wetting performance of the modified membranes can be indicated by the water vapor flux and scaling mass estimated in membrane distillation (MD) experiments. Direct contact MD (DCMD) with the concurrent flow was used in our experiments. Two custom-made cells with effective membrane areas of 12 cm^2^ and 32 cm^2^ were used in the flux experiment and scaling experiment, respectively. In the flux experiment, the feed stream was a 35 g·L^−1^ NaCl solution at a temperature of 60 °C, while DI water maintained at 20 °C was the distillate stream. The flow rates of the feed and distillate were maintained at 0.4375 cm s^−1^ using gear pumps, and the original volume of the feed and distillate solutions were 1800 mL and 800 mL, respectively. The scaling experiment was conducted under the same conditions, except that the feed solution was changed to a 20 mmol L^−1^ mixture of CaCl_2_ and Na_2_SO_4_ with the original volume of 600 mL. The mass and conductivity of the distillate were recorded constantly by an electronic scale and a conductivity meter, which determined the water vapor flux and indicated the salt concentration. After MD tests, the membrane was dried in the oven and weighed to calculate the weight difference compared to the original membrane.

The permeate flux (J, kg·m^−2^ h^−1^) and salt rejection (R) were calculated as follows [4,10,15]:

(1) $$\begin{array}{c} J=\frac{\Delta M}{A\cdot \Delta t} \end{array}$$

(2) $$\begin{array}{c} R=\left(1-\frac{C_{p}}{C_{f}}\right)\times 100\% \end{array}$$

where ΔM is the mass of the distillate water (kg), A is the effective membrane area (m^2^), Δt is the permeation time, $C_{p}$ and $C_{f}$ represent the salt concentrations of the permeate and feed, respectively. The volume change has been taken into account when calculating the $C_{p}$ and $C_{f}$.

## 3. Results and Discussion

### 3.1. Optimization of Plasma Treatment

Among the three different plasma treatment powers in our preliminary experiment, modification with 150 W plasma treatment power exhibited the highest water contact angle (WCA) (Table S1) and porosity. These results could be due to the higher plasma power, which would destroy more chemical bonds on the membrane surface, leading to larger pore size and membrane porosity. MD tests demonstrated that the 150 W treated membrane had reasonable vapor flux and good scaling resistance compared to other treated membranes (Figures S3 and S4). In addition, after the scaling MD tests, all the treated membranes exhibited lower scale deposition on the membrane surface than the pristine one, possibly due to their higher WCAs that increased the scale nucleation energy barrier [2] (Table S2 and Figure S5). Therefore, plasma-treated at 150 W was selected as the optimal power and applied in the following two modification strategies.

### 3.2. Membrane Characterizations

**Figure 2:**
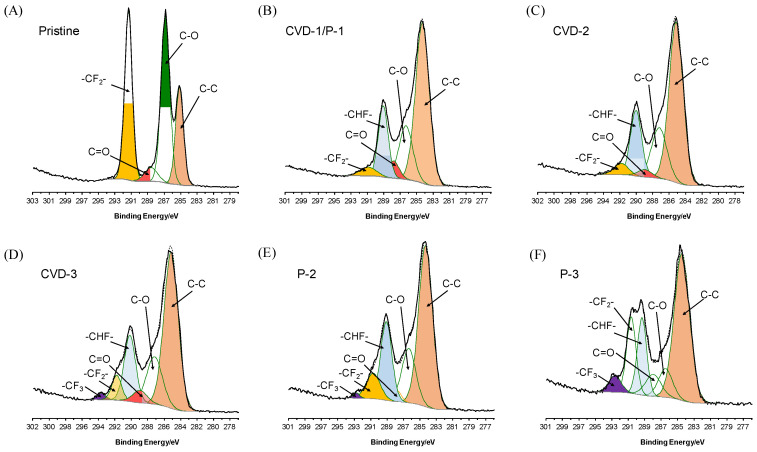
XPS spectra of C 1s binding energy of the pristine and modified membranes.

According to the SEM and elemental mapping images of the pristine and modified membranes (Figures S1 and S2), no significant distinction is observed on all the membranes, suggesting that the plasma-CVD (P-C) cycle had little effect on the membrane morphology. The Si element is detected on all modified membranes by SEM-EDS mapping and XPS analysis, while it does not exist on the pristine membrane. These results show that the fluorosilane was successfully grafted on the membrane surface (Table 1). To identify the surface composition difference obtained by different P-C cycles, the XPS spectrum of C 1s binding energy was further analyzed (Figure 2). For the pristine PVDF membrane, C 1s peaks located at 284.8 eV and 291.0 eV are assigned to the C-C bond and -CF_2_- bond, respectively (Figure 2A). The C-O and C=O bonds observed on the pristine PVDF could have resulted from the additives in the commercial membrane and the absorbed CO_2_. After one P-C cycle, the C 1s peaks exhibit significant change compared to the pristine one (Figure 2B). A new C 1s peak located at 289.5 eV is observed on the CVD-1/P-1, which could be assigned to the C-F bond formed during the plasma treatment process. In addition, the relative peak area for the -CF_2_- bond decreased significantly on the CVD-1/P-1, indicating that the 17-FAS-S density was relatively low after one CVD modification cycle. As the CVD cycle increased, the relative peak area for the -CF_2_- increased, showing that the 17-FAS-S modification density was increased by repeating the CVD procedure. Besides, a new peak is observed at 293.3 eV, which could be associated with the -CF_3_. Since -CF_3_ only appears at the end of the 17-FAS-S molecular chain, this observation further confirms that multiple CVD cycles could increase the modification density. On the other hand, repeating the P-C cycle, i.e., plasma treatment was conducted before each CVD step (P-2 and P-3), resulted in larger -CF_2_- and -CF_3_ peak areas (Figure 2E,F). Plasma treatment would break the chemical bonds and generate new reaction sites on the membrane surface and the previously modified 17-FAS-S molecules, allowing more 17-FAS-S to be grafted. Therefore, the P-C cycle would result in a more branched surface structure. Based on the relative peak area of -CF_3_, it is clear that the surface fluorination layer on the P-3 had the highest branch level.

To further characterize the membranes, the pore size, WCA, and porosity of all membranes were tested. As mentioned in Section 3.1, after one P-C cycle, the resulted membrane (CVD-1/P-1) showed an enlarged pore size and porosity compared to the pristine one. However, the membrane pore size decreased as the CVD or P-C cycle was repeated (Table 2). It is speculated that both CVD and P-C treatment strategies could graft more FAS on the membrane surface, which may shrink the membrane pore structure due to a denser coating layer. On the other hand, no distinct difference was observed in the WCA with the increased CVD times, suggesting that increasing the FAS surface density is marginal to the apparent WCA. Classic contact angle theory suggests that the apparent contact angle is determined by material surface energy and surface roughness. Repeating the CVD process would increase the FAS modification density; however, the corresponding surface energy may not have a significant change during this CVD cycle because the reactive sites generated by plasma treatment are nearly saturated after the first CVD cycle. Besides, 17-FAS-S modification is a monolayer coating, which means the CVD processes would not affect the surface roughness. Consequently, the WCAs of CVD-1/P-1, CVD-2, and CVD-3 are similar. 

For the membranes treated with P-C cycles, the hydrophobicity improves with increased cycles, and P-3 was even close to superhydrophobicity (WCA > 150°). According to previous research, decorating the membrane surface with nanoparticles could render promising anti-wetting properties by increasing the surface roughness and offering more hydroxyl groups on the membrane surface to react with FAS [16,29]. For comparison, no nanoparticle was used in this study, and the increased WCA is mainly attributed to the branched FAS structure. As we discussed above, the repeated plasma treatment could provide more reactive sites for fluorination by breaking down the C-F bonds on the previously grafted 17-FAS-S molecules. The subsequent CVD process allows the new 17-FAS-S to graft on these active sites, forming branched structures. These branched structures increase the surface roughness at the molecular level, thus increasing the WCA (Table 2). 

### 3.3. MD Performance with NaCl

**Figure 3:**
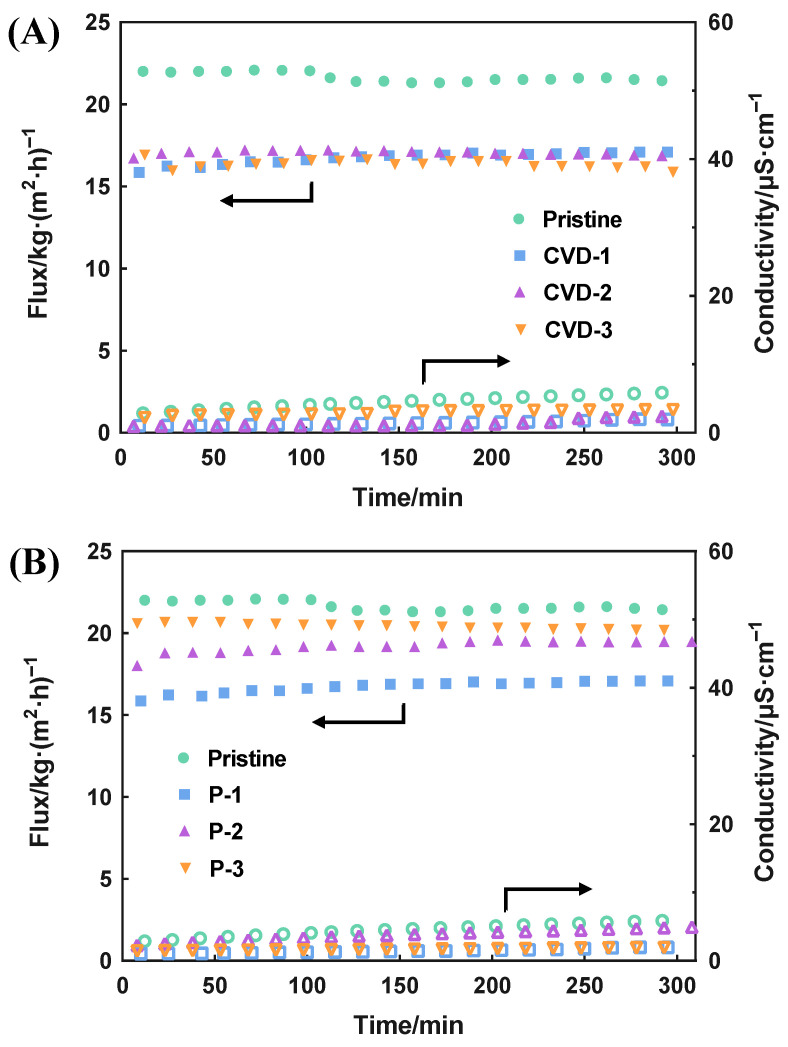
The MD performance of different modified times of (**A**) CVD and (**B**) P-C treatment (feed solution: 35 g·L^−1^ NaCl).

The performance of all the membranes was first tested using a DCMD setup with a NaCl feed solution. Figure 3 depicts the water vapor flux and permeate conductivity of the pristine and modified membranes. Without adding the scaling agent, the concentrated feed solution at the end of the test did not reach its saturated concentration at 60 °C. Therefore, no scaling phenomenon was observed during this experiment. The pristine membrane exhibited a stable flux of 21.67 ± 0.28 kg·m^−2^ h^−1^, while all the modified membranes showed declined fluxes in varying degrees. These results indicate that the modification has led to a certain flux compromise. All the membranes have stable performance in the MD tests, for the conductivities were steady within 10 μS·cm^−1^, which means the salt rejection was maintained above 99.98%.

However, there is a difference in the membrane flux between the CVD series membranes and P series membranes. All the CVD membranes (CVD-1, -2, and -3) showed almost identical flux throughout the tests, implying that increasing the FAS modification density has little effect on the vapor transport (Figure 3A). In contrast, repeating the P-C cycle exhibited increased membrane flux compared to CVD-1/P-1 (P-2 and P-3) (Figure 3B). This phenomenon indicates that the branched FAS structure could alleviate the flux decline effect caused by traditional FAS coating. We hypothesize that the low surface energy FAS with a branched structure might lower the Knudsen flow resistance, thereby improving the membrane flux [29]. The flux of P-3 was maintained at 20.42 ± 0.16 kg·m^−2^ h^−1^ with a salt rejection above 99.99%, illustrating its excellent performance compared to commercial PVDF membrane.

### 3.4. MD Performance with CaSO_4_

**Figure 4:**
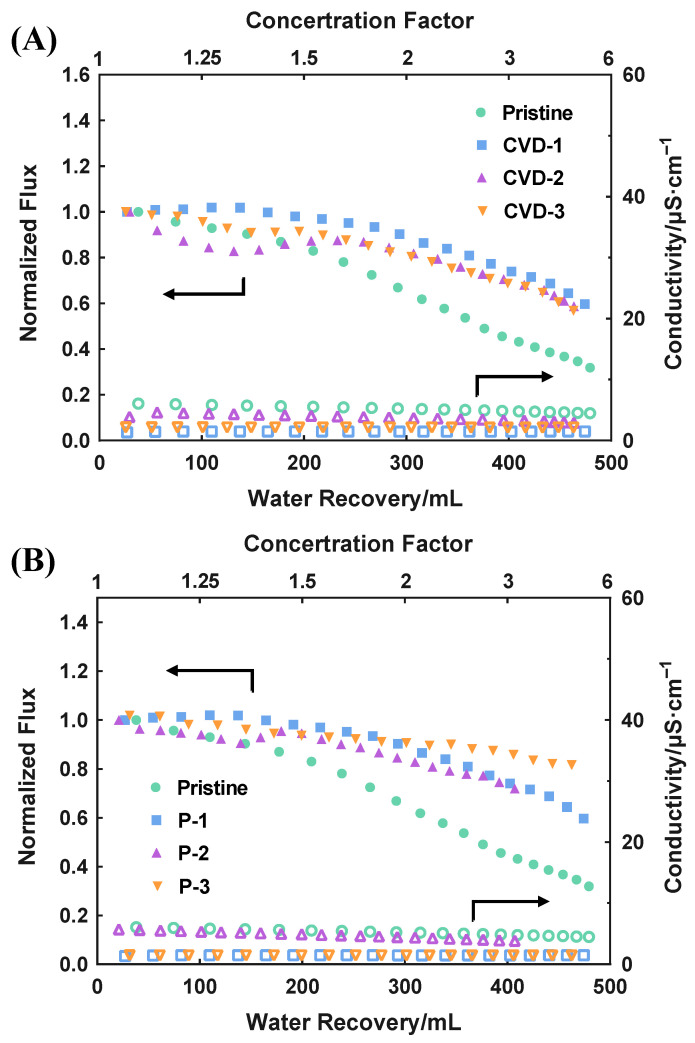
The MD performance of different modified times of (**A**) CVD and (**B**) P-C treatment (feed solution: 20 mM mixture of CaCl_2_ and Na_2_SO_4_). Initial flux, Pristine: 16 kg·m^−2^ h^−1^; CVD-1/P-1: 11 kg·m^−2^ h^−1^; CVD-2: 12 kg·m^−2^ h^−1^; CVD-3: 10 kg·m^−2^ h^−1^; P-2: 13 kg·m^−2^ h^−1^; P-3: 15 kg·m^−2^ h^−1^.

The MD performance with the scaling agent is depicted in Figure 4, in which the flux is presented in normalized flux (ratio of instantaneous flux to the initial flux) to better distinguish the flux variation. Unlike the stable flux in MD experiments with NaCl, the flux of all membranes declined to a different extent as the water recovery increased. The feed solution was concentrated with a concentration factor of 6 at the end of the test. Considering the combined effect of concentration and temperature polarizations [31,32], the membrane surface tends to have a higher saturation index (SI), resulting in a higher potential for scale formation [2]. Therefore, all the tested membranes exhibited flux decline during the experiment due to the gypsum scales deposited on the membrane surface and blocking the membrane pores. Moreover, the scaling did not penetrate the membranes since no sharp increase in permeate conductivity was observed for all the membranes. 

All the modified membranes exhibited mitigation of flux decline compared to the pristine PVDF, showing that both the CVD and P-C strategies are effective for scaling resistance improvement. Among them, the P-3 membrane maintained about 80% of its initial flux at the end of the test, which outperforms other modified membranes (Figure 4B). To further evaluate the scaling status, scales formed on the membrane surface were weighed and listed in Table 3. The scale mass is negligible on the membrane surface of CVD-3, P-2, and P-3, much smaller than that on the pristine PVDF. This result further confirms their improved scaling resistance. 

**Figure 5:**
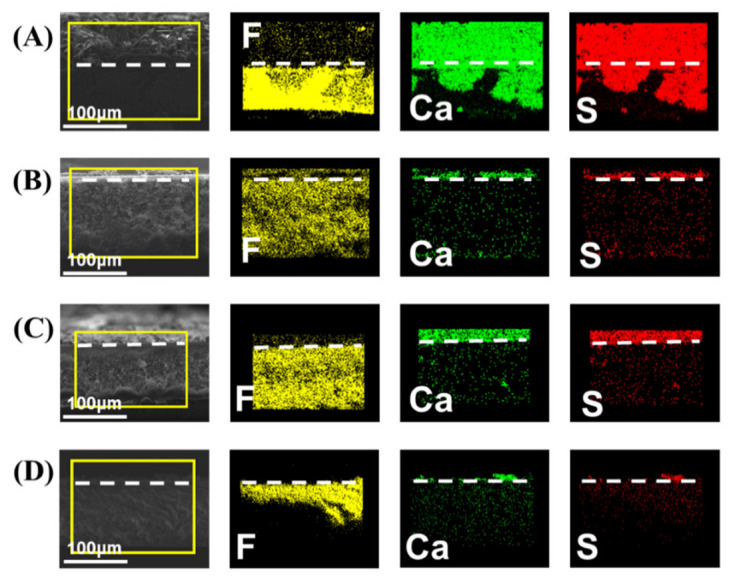
The cross-section of SEM and elemental mapping images of (**A**) pristine, (**B**) CVD-1, (**C**) CVD-2, and (**D**) CVD-3.

**Figure 6:**
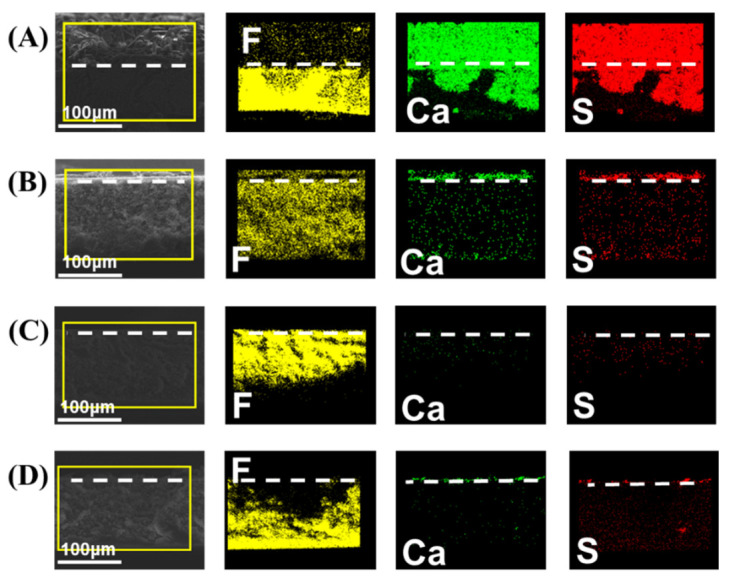
The cross-section of SEM and elemental mapping images of (**A**) pristine, (**B**) P-1, (**C**) P-2, and (**D**) P-3.

Cross-section SEM and SEM-EDS elemental mapping were used to inspect the scale intrusion to the membranes (Figure 5 and Figure 6). From the element images of Ca and S, representing the gypsum scale, it is evident that the gypsum crystals had deeply intruded into the pristine membrane. For comparison, no significant scale intrusion was observed on other membranes, and only a small amount of crystals were observed on the surface of the modified membranes. These results are consistent with the MD tests and demonstrate that P-3 has the best anti-scaling performance in initial flux and scaling mitigation. 

### 3.5. Comprehensive Analysis and Discussion

The exceptional anti-scaling performance in MD experiments of P-3 membrane can be explained by the following aspects. For the scaling resistance, first of all, the large WCA of P-3 results in a small liquid−solid contact interface on the membrane surface, reducing the available area for crystal deposition and suppressing the heat diffusion to alleviate temperature polarization [31,32]. Secondly, according to the classic nucleation theory (CNT), the energy barrier of heterogeneous nucleation is negatively associated with the membrane surface energy [33]. Therefore, the high density and uniformity of fluorination in P-3 endow the membrane with very low surface energy, inhibiting the heterogeneous nucleation and reducing the possibility of crystal adhesion [2,34]. Besides the thermodynamic argument, there is also a hydrodynamic consideration. What we used to modify the membrane surface is proven to be a liquid-like coating [29], providing a slippery boundary condition for liquid flow along the membrane surface [30,34]. With a higher flow velocity and turbulence intensification near the membrane surface, the concentration polarization will be mitigated, thus preventing nucleation. 

As indicated by the XPS result (Figure 2F), P-3 had the highest branch level. Combined with the small pore size, it is clear that the P-3 has the maximal fluorination density and the highest surface roughness in a molecular level, resulting in a superior ability to maintain a Cassie−Baxter state [30]. All the above effects contribute to the excellent performance of P-3.

## 4. Conclusions

This study proposed an effective surface modification strategy for commercial PVDF membranes by manipulating the intensity and times of the plasma-CVD treatment to control the fluorination density. Comparing the surface properties of the membranes obtained in the two different approaches, it is indicated that repeating the P-C cycle could increase the fluorination density and the surface molecule branch level. In combination with the MD experiments, the P-3 membrane has shown remarkable anti-scaling capability. To conclude, employing the P-C modification to commercial PVDF membrane can significantly improve its anti-scaling property with a more stable and higher flux in a strong scaling tendency situation. As a facile and satisfactory method, it has a wide prospect of application to endow traditional hydrophobic membranes with better behavior in the MD process. Moreover, with further exploration of the specific mechanism, this modification is scalable for other membrane substrates and has the potential to substitute the direct utilization of nanoparticles with uncompromised flux in commercial desalination. 

## Figures and Tables

**Table 1:** The element ratio of different CVD and plasma treatment times modified membranes.

Membrane Types	C/%	O/%	F/%	Si/%
Pristine	41.1	1.72	57.18	0
CVD-1/P-1	47.55	1.3	51.09	0.05
CVD-2	39.16	2.11	58.63	0.1
CVD-3	47.10	1.86	51.00	0.04
P-2	46.13	1.85	51.98	0.03
P-3	45.52	2.51	51.82	0.15

**Table 2:** The membrane characterization of different CVD and plasma treatment times for modified membranes.

Membrane	Pore Size μm	Water Contact Angle °	Porosity %	Bubble Point μm
Pristine	0.28 ± 0.009	131.1 ± 1.3	59.51 ± 2.65	0.426
CVD-1/P-1	0.43 ± 0.025	140.4 ± 1.1	77.77 ± 1.64	0.447
CVD-2	0.32 ± 0.023	142.2 ± 1.1	75.72 ± 3.57	0.440
CVD-3	0.30 ± 0.008	141.1 ± 1.7	78.97 ± 4.64	0.422
P-2	0.42 ± 0.009	142.4 ± 2.1	70.79 ± 3.07	0.444
P-3	0.29 ± 0.009	149.5 ± 1.3	73.39 ± 1.95	0.340

**Table 3:** The scale mass on the membrane surface after scaling tests.

Membrane Types	Pristine	CVD-1/P-1	CVD-2	CVD-3	P-2	P-3
Scale Mass/g	0.1792	0.0244	0.0160	0.0048	0.0056	0.0058

## Supplementary Materials

The following supporting information can be downloaded at: https://www.mdpi.com/article/10.3390/membranes12080743/s1. Figure S1: The SEM and Mapping images of (A) Pristine, (B) CVD-1, (C) CVD-2, and (D) CVD -3; Figure S2: The SEM and Mapping images of (A) Pristine, (B) P-1, (C) P-2, and (D) P-3; Figure S3: The MD performance of different modified power of plasma treatment. (Feed solution: 35 g·L^−1^ NaCl); Figure S4: The MD performance of different modified power of plasma treatment (Feed solution: 20 mM mixture of CaCl_2_ and Na_2_SO_4_); Figure S5: Scaling on pristine, 50 W, 100 W, and 150 W membrane after MD test; Figure S6: Scaling on Pristine, CVD-1, CVD-2, and CVD-3 membrane after MD test; Figure S7: Scaling on pristine, P-1, P-2, and P-3 membrane after MD test; Table S1: The membrane characterization of different plasma power modified membranes; Table S2: The scaling mass on different plasma power modified membrane surface.
