# Supplementary material for: Multi-Layered Branched Surface Fluorination on PVDF Membrane for Anti-Scaling Membrane Distillation
Source: Membranes (Basel). 2022 Jul 29;12(8):743. doi: 10.3390/membranes12080743 (PMC9416731; doi:10.3390/membranes12080743)
Supplement: Supplementary file 1 [file membranes-12-00743-s001.zip › membranes-1821514-supplementary.pdf]

membranes

Supplementary Materials

Multi-layered Branched Surface Fluorination on PVDF Membrane for Anti-scaling Membrane Distillation

Yu-Jing Liu [1], Yan-Nan Lu [1], Dong-Qing Liang [1,2], Yin-Shuang Hu [1,2] and Yu-Xi Huang [1,2,*]

[1] School of Environmental Science and Engineering, Sun Yat-sen University, Guangzhou, Guangdong 510275, China
[2] Guangdong Provincial Key Laboratory of Environmental Pollution Control and Remediation Technology (Sun Yat-sen University), Guangzhou 510275, PR China
* Correspondence: Yu-Xi Huang, E-mail: huangyx253@mail.sysu.edu.cn

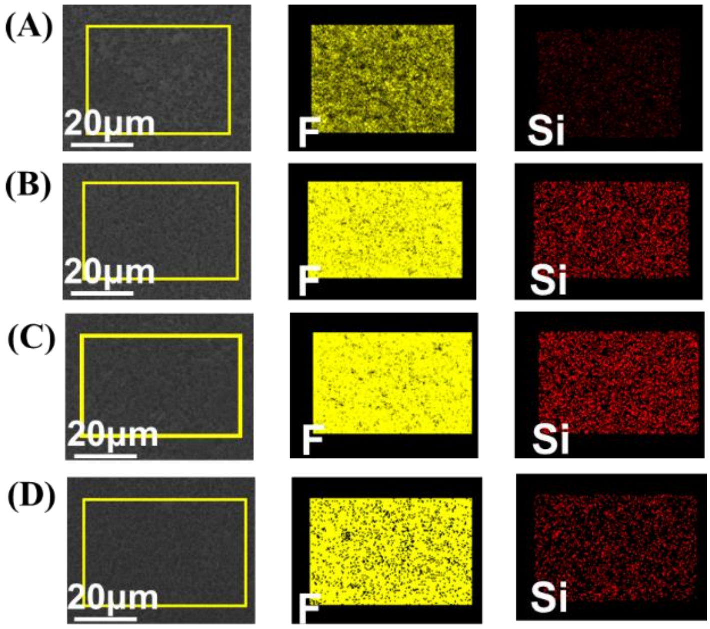

Figure S1. The SEM and Mapping images of.(A) Pristine (B) CVD-1 (C) CVD-2 (D) CVD -3.

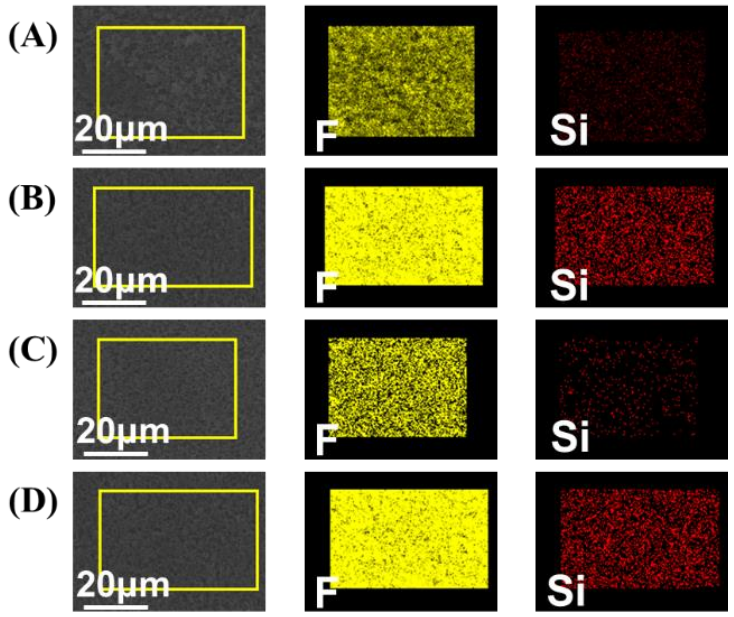

Figure S2. The SEM and Mapping images of (A) Pristine (B) P-1 (C) P-2 (D) P-3.

Table S1. The membrane characterization of different plasma power modified membranes.

Membrane Types	Pore Size/μm	Water Contact Angle/°	Porosity/%	Bubble Point/μm
Pristine	0.28	131.1	59.51	0.426
50W	0.29	125.8	68.62	0.326
100W	0.40	133.9	71.61	0.435
150W	0.43	140.4	77.77	0.447

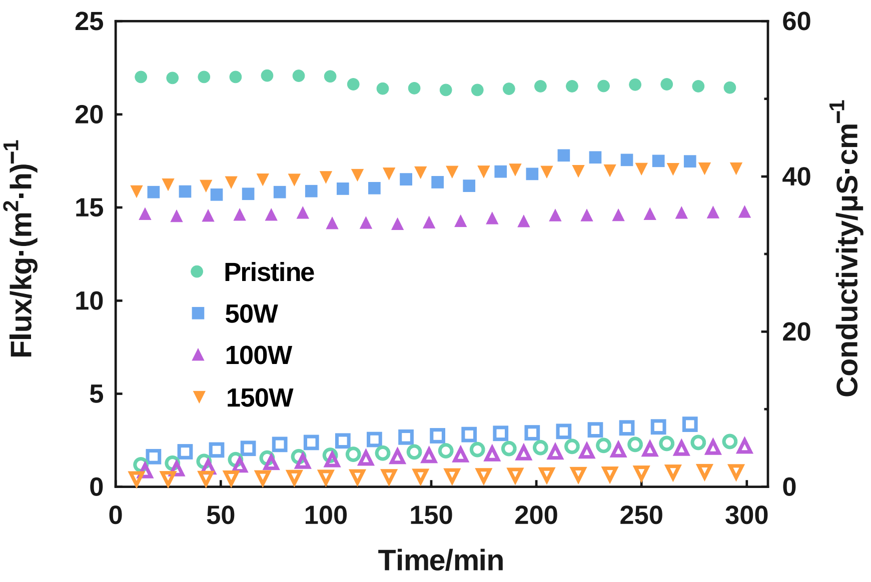

.

Figure S3. The MD performance of different modified power of plasma treatment. (Feed solution: 35g·L^{-1} NaCl).

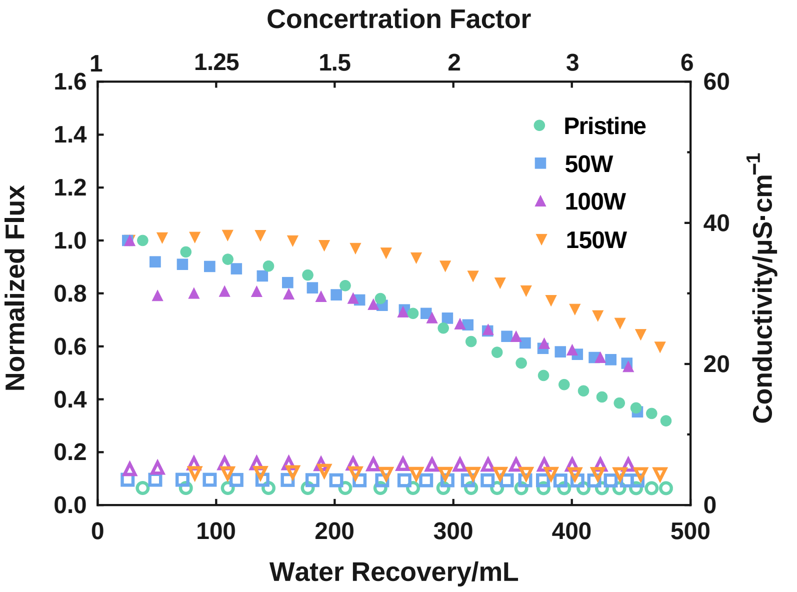

Figure S4. The MD performance of different modified power of plasma treatment(Feed solution: 20mM mixture of $CaCl_2$ and Na_2SO_4).

Table S2. The scaling mass on different plasma power modified membrane surface.

Membrane Types	Pristine	50W	100W	150W
Scaling Mass /g	0.1792	0.0412	0.0405	0.0244

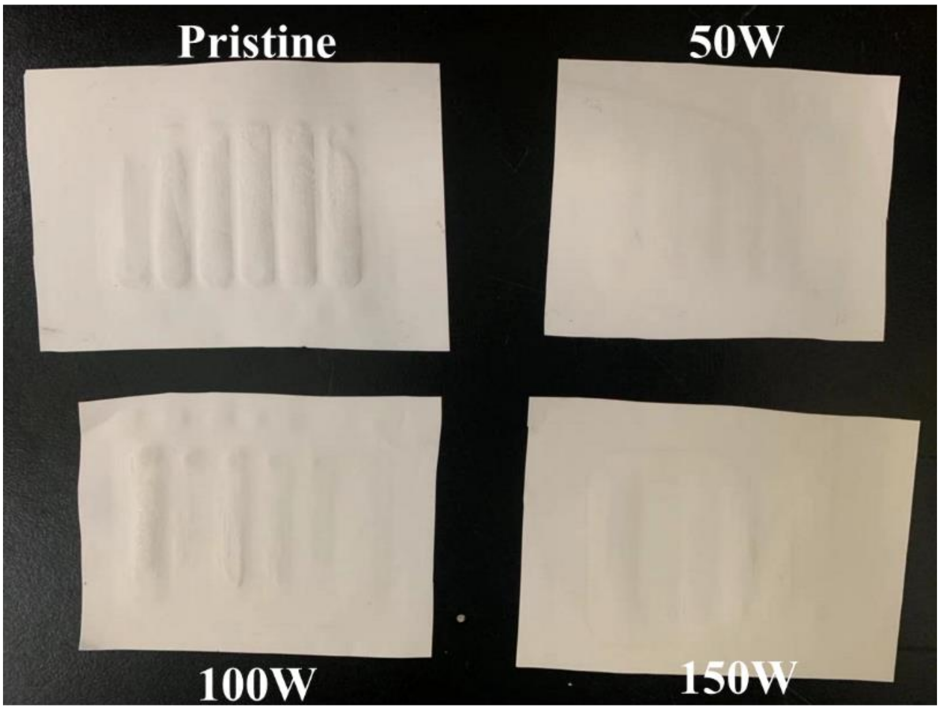

Figure S5. Scaling on Pristine, 50 W, 100 W, and 150 W membrane after MD test.

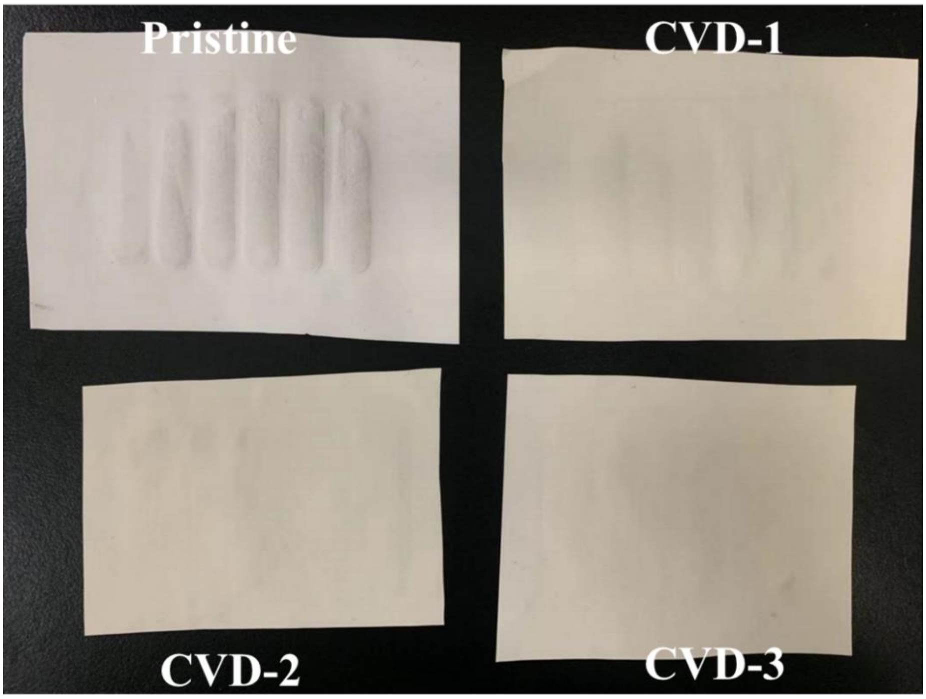

Figure S6. Scaling on Pristine, CVD-1, CVD-2 and CVD-3 membrane after MD test.

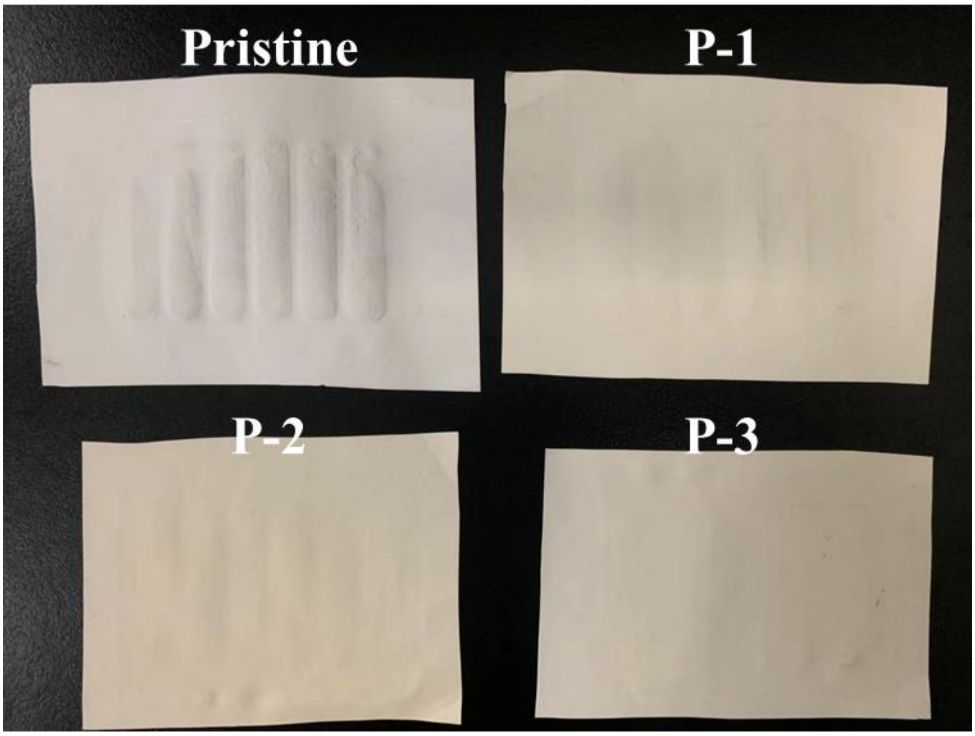

Figure S7. Scaling on Pristine, P-1, P-2 and P-3 membrane after MD test.
